# Potential molecular and cellular mechanisms of the effects of cuproptosis-related genes in the cardiomyocytes of patients with diabetic heart failure: a bioinformatics analysis

**DOI:** 10.3389/fendo.2024.1370387

**Published:** 2024-05-31

**Authors:** Jinhao Chen, Xu Yang, Weiwen Li, Ying Lin, Run Lin, Xianzhen Cai, Baoxin Yan, Bin Xie, Jilin Li

**Affiliations:** ^1^ Department of Cardiology, Second Affiliated Hospital of Shantou University Medical College, Shantou, Guangdong, China; ^2^ Shantou University Medical College, Shantou, Guangdong, China

**Keywords:** cuproptosis, diabetic heart failure, fibrosis, exosome, immune regulation

## Abstract

**Background:**

Diabetes mellitus is an independent risk factor for heart failure, and diabetes-induced heart failure severely affects patients’ health and quality of life. Cuproptosis is a newly defined type of programmed cell death that is thought to be involved in the pathogenesis and progression of cardiovascular disease, but the molecular mechanisms involved are not well understood. Therefore, we aimed to identify biomarkers associated with cuproptosis in diabetes mellitus-associated heart failure and the potential pathological mechanisms in cardiomyocytes.

**Materials:**

Cuproptosis-associated genes were identified from the previous publication. The GSE26887 dataset was downloaded from the GEO database.

**Methods:**

The consistency clustering was performed according to the cuproptosis gene expression. Differentially expressed genes were identified using the limma package, key genes were identified using the weighted gene co-expression network analysis(WGCNA) method, and these were subjected to immune infiltration analysis, enrichment analysis, and prediction of the key associated transcription factors. Consistency clustering identified three cuproptosis clusters. The differentially expressed genes for each were identified using limma and the most critical MEantiquewhite4 module was obtained using WGCNA. We then evaluated the intersection of the MEantiquewhite4 output with the three clusters, and obtained the key genes.

**Results:**

There were four key genes: *HSDL2*, *BCO2*, *CORIN*, and *SNORA80E*. *HSDL2*, *BCO2*, and *CORIN* were negatively associated with multiple immune factors, while *SNORA80E* was positively associated, and T-cells accounted for a major proportion of this relationship with the immune system. Four enriched pathways were found to be associated: arachidonic acid metabolism, peroxisomes, fatty acid metabolism, and dorsoventral axis formation, which may be regulated by the transcription factor MECOM, through a change in protein structure.

**Conclusion:**

HSDL2, BCO2, CORIN, and SNORA80E may regulate cardiomyocyte cuproptosis in patients with diabetes mellitus-associated heart failure through effects on the immune system. The product of the cuproptosis-associated gene *LOXL2* is probably involved in myocardial fibrosis in patients with diabetes, which leads to the development of cardiac insufficiency.

## Background

Diabetes mellitus (DM) is an important risk factor for heart failure (1). Epidemiological and observational studies have shown that prolonged diabetes causes structural and functional changes in the heart, leading to heart failure, and this process is independent of myocardial ischemia or microvascular atherosclerotic disease (2). In other words, this etiology is associated with a distinct type of pathology. The prevalence of heart failure has been shown to be as high as 14.5% in patients with type 1 diabetes mellitus (T1DM) and 35% in those with type 2 diabetes mellitus (T2DM) (3, 4). Each 1% increase in glycated hemoglobin A1c is associated with a 30% and 8% increases in the risk of heart failure in people with T1DM and T2DM, respectively (5). The Framingham Heart Study of 5,209 men and women who were followed for 18 years showed that diabetic patients are at a high risk of new-onset and recurrent heart failure (6). The pathogenesis of heart failure in diabetes mellitus is complex and has not yet been fully characterized. However, it has been suggested that the translocation of CD36 to the myocardium, activation of NLRP3 inflammatory vesicles, upregulation of the AGE/RAGE system, and microRNA imbalance induce oxidative stress/inflammation-associated myocardial remodeling and ventricular dysfunction in patients with diabetes mellitus in the context of dysglycemia (7).

Cuproptosis occurs through the direct binding of copper ions to lipoylated components of the tricarboxylic acid cycle (TCA), which leads to the aggregation of lipoylated proteins, with iron-thioglycosan-containing proteins causing a decrease in the expression of iron-thioglycosan, which triggers proteotoxic stress and ultimately cell death (8). In recent years, cuproptosis has been increasingly recognized to be an important mediator of the pathogenesis and progression of cardiovascular disease, which involves reactive oxygen species (ROS) accumulation, proteasome inhibition, and mitochondrial dysfunction (9). Notably, in diabetic heart failure, copper metabolism in the myocardium has been shown to be abnormal (10). Moreover, animal studies have shown that the administration of copper chelators restores the structure and function of the myocardium in diabetic rats by correcting abnormalities in factors involved in the assembly of copper-coated proteins and cytochrome oxidase (11). However, the authors of this study did not identify a specific mechanism for this.

Recent cell and mouse studies have demonstrated that excess advanced glycosylation end-products (AGEs) and copper in patients with diabetes cause the upregulation of ATF3/SPI1/SLC31A1 signaling, leading to downregulation of the mitochondrial respiratory chain complex, a reduction in ATP production, and inhibition of the activity of mitochondrial complexes I and III, resulting in cardiac cytotoxicity (12–14). Thus, there appear to be relationships among cuproptosis, diabetes, and heart failure. In a meta-analysis of data derived from 1,504 people, a significant association between high serum copper concentration and heart failure was identified (15). In addition, a high serum copper/zinc ratio has been shown to be associated with a higher risk of heart failure in middle-aged and older Finnish men (16).

High plasma concentrations of cuprocyanin (CER), which accounts for >95% of the total plasma copper content, are associated with a higher risk of heart failure and a poor prognosis (17). Several issues are worth considering. Firstly, although a high CER appears to be associated with higher mortality in patients with heart failure, a compensatory increase in CER appears to have protective and antioxidant effects (18). Secondly, previous studies have demonstrated the involvement of CER in iron metabolism, including the conversion of Fe^2+^ to Fe^3+^ and the prevention of the Fenton reaction (19); and G6PD is involved in the regulation of both iron-related cell death and cuproptosis-related genes (20), implying that CER is involved in redox regulation. However, several previous studies have shown that CERs have not only antioxidant, but also pro-oxidant, properties that are environmentally dependent (21). This suggests that changes in serum copper concentration and their effects may involve regulation at the genomic level. Therefore, it is important to explore the potential role of cuproptosis-related genes in the cardiomyocytes of patients with diabetes mellitus in combination with heart failure.

In the present study, we aimed to identify the differences in expression and corresponding functional enrichment of cuproptosis-related genes in cardiomyocytes from patients with diabetes mellitus-associated heart failure to explore the cuproptosis-related molecular mechanisms in patients with diabetes mellitus in combination with heart failure and to identify potential regulatory genes.

## Materials

### Data download

We searched the NCBI GEO Public Database for expression matrices associated with diabetic heart failure, including datasets collected from clinical studies. The GSE26887 dataset, which had an annotation platform of GPL6244, was downloaded from the GEO database (https://www.ncbi.nlm.nih.gov/geo/). This comprised data for a total of 19 samples from human myocardial tissue (12 healthy control samples and 7 disease samples). A list of diabetic heart failure-related genes from the GeneCards database (https://www.genecards.org/) was selected as the validation set. Cuproptosis-associated genes were identified from the previous publication “*Identification of cuproptosis-related subtypes and development of a prognostic signature in colorectal cancer*.” (DOI: 10.1038/s41598-022-22300-2).

### Consistent clustering of cuproptosis genes

Based on the expression of cuproptosis genes, the subtypes of diabetic heart failure cuproptosis were clustered using the consistency clustering method in the R (R Foundation for Statistical Computing, Vienna, Austria) package ConsensusClusterPlus (version 1.66.0-1) (adjusted *p* < 0.05) using 50 iterations, with each iteration containing 80% of the samples. The optimal number of clusters was determined using the cumulative distribution function curves of the consistency scores and the features of the consistency matrix heat map.

### Analysis of immune cell infiltration

The data were analyzed using the CIBERSORT algorithm (version 1.03), which was used to infer the relative proportions of 22 types of infiltrating immune cells and construct immune cell histograms, correlation heatmaps, and box-and-line plots; as well as to perform correlation analyses of the relationships between the expression of key genes and immune cells.

### Differential expression analysis

The R package “limma” (version 3.52.4) was used to identify the molecular mechanisms of each subtype from the diabetic heart failure data and to identify differentially expressed genes, using the criteria |logFC| > 0.585 and *P* < 0.05. Volcano maps of the differentially expressed genes were drawn.

### WGCNA network construction

Weighted gene co-expression networks were constructed to search for co-expressed gene modules and to explore the relationship between gene networks and various subtypes, as well as with the key genes in the networks. The co-expression network was constructed using the WGCNA-R package (version 1.71), with the soft threshold being set to 17. The weighted neighbor-joining matrix was transformed into a topological overlap matrix (TOM) to estimate the network connectivity, and the hierarchical clustering method was used for the clustering tree structure of the TOM matrix. The various branches of the clustering tree represented the various gene modules and the differing colors represented the different modules. Based on the weighted correlation coefficients for the genes, they were classified according to their expression patterns, and genes with similar patterns were placed into a single module. In this way, tens of thousands of genes were allocated to multiple modules using their expression patterns.

### lncRNA-miRNA-mRNA network construction

Competitive endogenous RNAs (ceRNAs) have been identified to be a novel means of regulating gene expression. ceRNA regulatory networks are more refined and complex than miRNA regulatory networks; involve more RNA molecules; and include mRNAs, pseudogenes coding for genes, long-chain non-coding RNAs and miRNAs, and circRNAs. Mircode (http://www.mircode.org/) is a database that is commonly used to interrogate relationships between lncRNAs and miRNAs and covers the complete GENCODE-annotated transcriptome, including non-coding RNA genes with lengths of >10,000 bases. In the present study, the Mircode database was used to predict lncRNA-miRNA interaction pairs, then a lncRNA-miRNA-mRNA network was constructed by combining the mRNA-miRNA and lncRNA-miRNA interactions, and visualized using Cytoscape (version 3.9.1).

### GSEA pathway enrichment analysis

GSEA analysis uses a predefined set of genes (KEGG) to rank genes according to their level of differential expression in two types of samples, and then evaluates whether a predefined set of genes is at the top or bottom of this ranking. In the present study, by using “clusterProfiler” R package (version 4.4.4) (adjusted p < 0.05), we explored the potential molecular mechanisms associated with the genes characterizing the GSEA comparison group and the low-expression group by evaluating the differences in signaling pathways between the two groups, with the number of substitutions set to 1,000 and the type of substitution set to phenotype.

### Analysis of the transcriptional regulation of key genes

We predicted the transcription factors involved in the molecular mechanisms using the R package “RcisTarget” (version 1.2.1), which uses calculations based on gene-motif rankings and the annotation of motifs to transcription factors. The normalized enrichment score (NES) of a motif depends on the total number of motifs in the database. In addition to the motifs annotated in the source data, we inferred further annotation files on the basis of motif similarity and gene sequence. The first step in estimating the overexpression of each motif in a gene set is to calculate the area under the curve (AUC) for each motif-motif set pair. This was performed using the recovery curve constructed for the gene-set-to-motif sequencing. The NES for each motif was calculated on the basis of the AUC distribution of all the motifs in the gene set.

### Statistical analysis

Statistical analyses were performed using the R language (R Foundation for Statistical Computing, Vienna, Austria) (version 4.2.2) and the findings were accepted as being statistically significant when *p*<0.05.

## Results

### Differential expression of cuproptosis-related genes in the myocardial tissue of patients with diabetic heart failure vs. normal individuals

**Figure 1:**
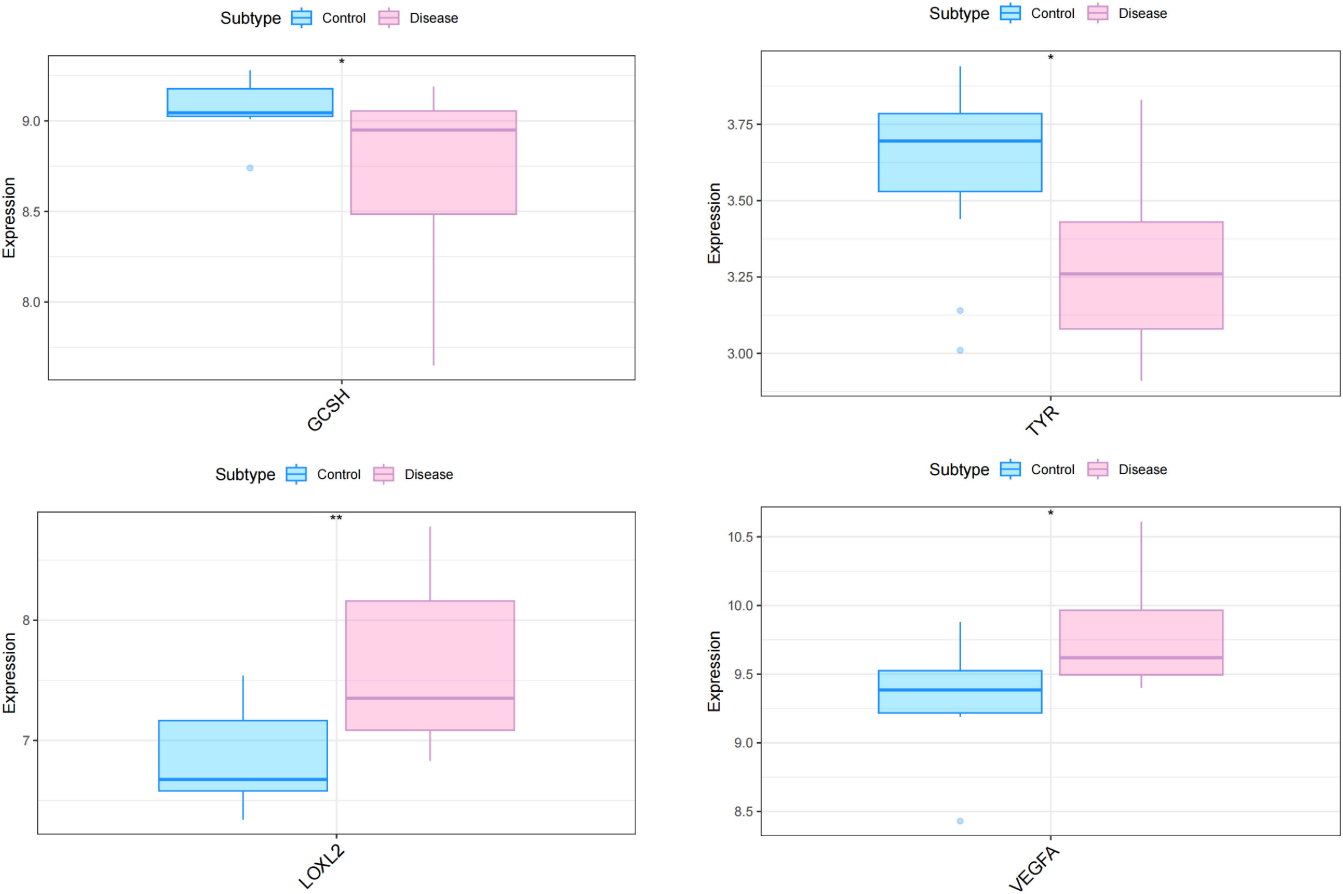
Differential expression of four cuproptosis-related genes in the myocardial tissue of patients with diabetes and heart failure vs. healthy controls. The expression of *GCSH* and *TYR* was lower in the disease samples, whereas that of *LOXL2* and *VEGFA* was higher. *p<0.05; **p<0.01.

**Figure 2:**
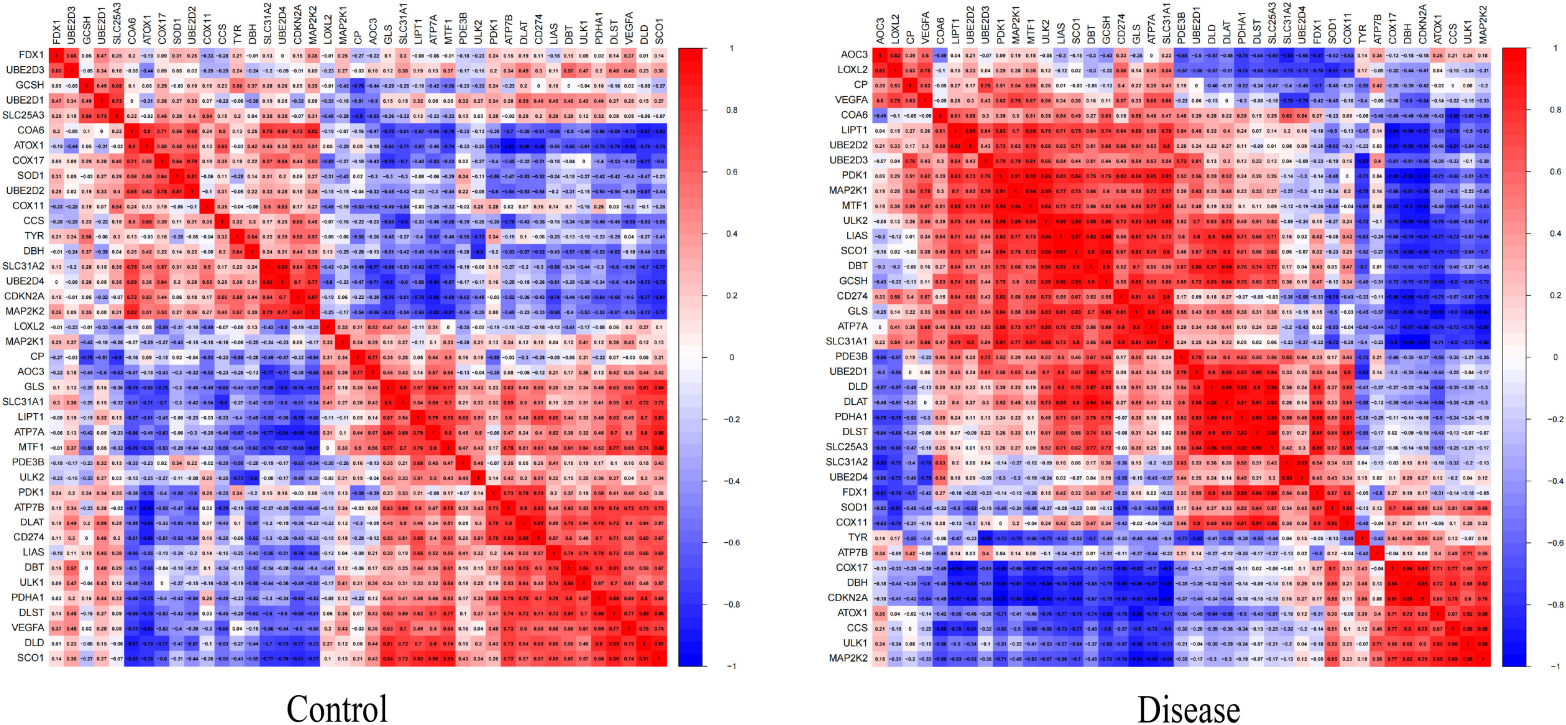
Heat map of the differentially expressed genes associated with cuproptosis between samples from patients with diabetic heat failure and healthy controls. The proportion of significant pairings between cuproptosis genes was significantly higher in the disease group than in the control group.

To explore whether the pattern of cuproptosis-related gene expression in diabetic heart failure could explain the heterogeneity of the disease in patients, we used 19 samples from GSE26887 (12 healthy control samples and 7 diabetic heart failure samples). Forty-four cuproptosis-related genes were identified in the literature, and this list was narrowed to the 41 genes available in GSE26887. We then analyzed the expression of these cuproptosis-related genes in the normal and diabetic heart failure samples, and found that four were differentially expressed: *GCSH* and *TYR* expression was lower in the disease samples, and *LOXL2* and *VEGFA* expression was higher (**Figure 1**) (**Supplementary Table 1**). In addition, we performed correlation analyses of the cuproptosis-related genes in the control and disease samples, and identified multiple significant relationships between cuproptosis gene pairs (**Figure 2**) (**Supplementary Tables 2**, **3**).

### LOXL2 expression differs significantly according to the subtype of cuproptosis-related gene expression in patients with diabetic heart failure

**Figure 3:**
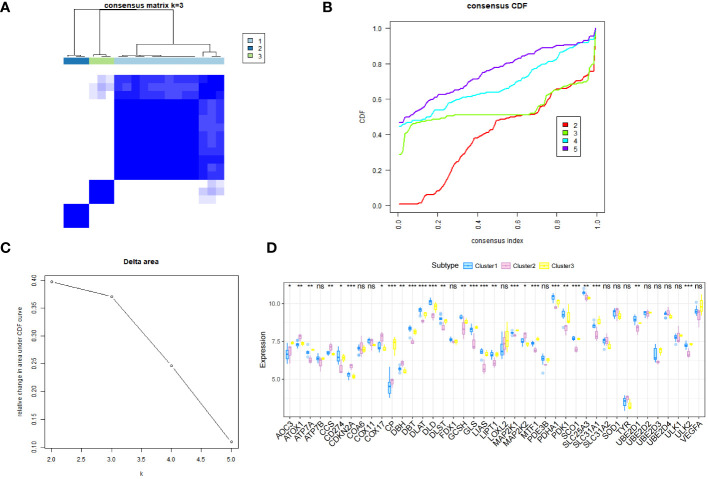
The samples in the GSE26887 dataset were allocated to three subtypes. **(A)** Hierarchical clustering of the genes. Based on the expression of the cuproptosis-related genes, the diabetic heat failure genes were clustered into three modules. **(B)** Consistent cumulative distribution function graph, showing that a clustering number (K) of 3 was optimal. **(C)** Delta area plot, showing that a K of 3 was optimal. **(D)** Differences in the expression of cuproptosis-related genes among the three subtypes. The inter-subtype difference in *LOXL2* expression was the largest of the four differentially expressed genes (*GCSH*, *TYR*, *LOXL2*, and *VEGFA*). *, p<0.05; **, p<0.01; ***, p<0.001; ns, no statistically.

**Figure 4:**
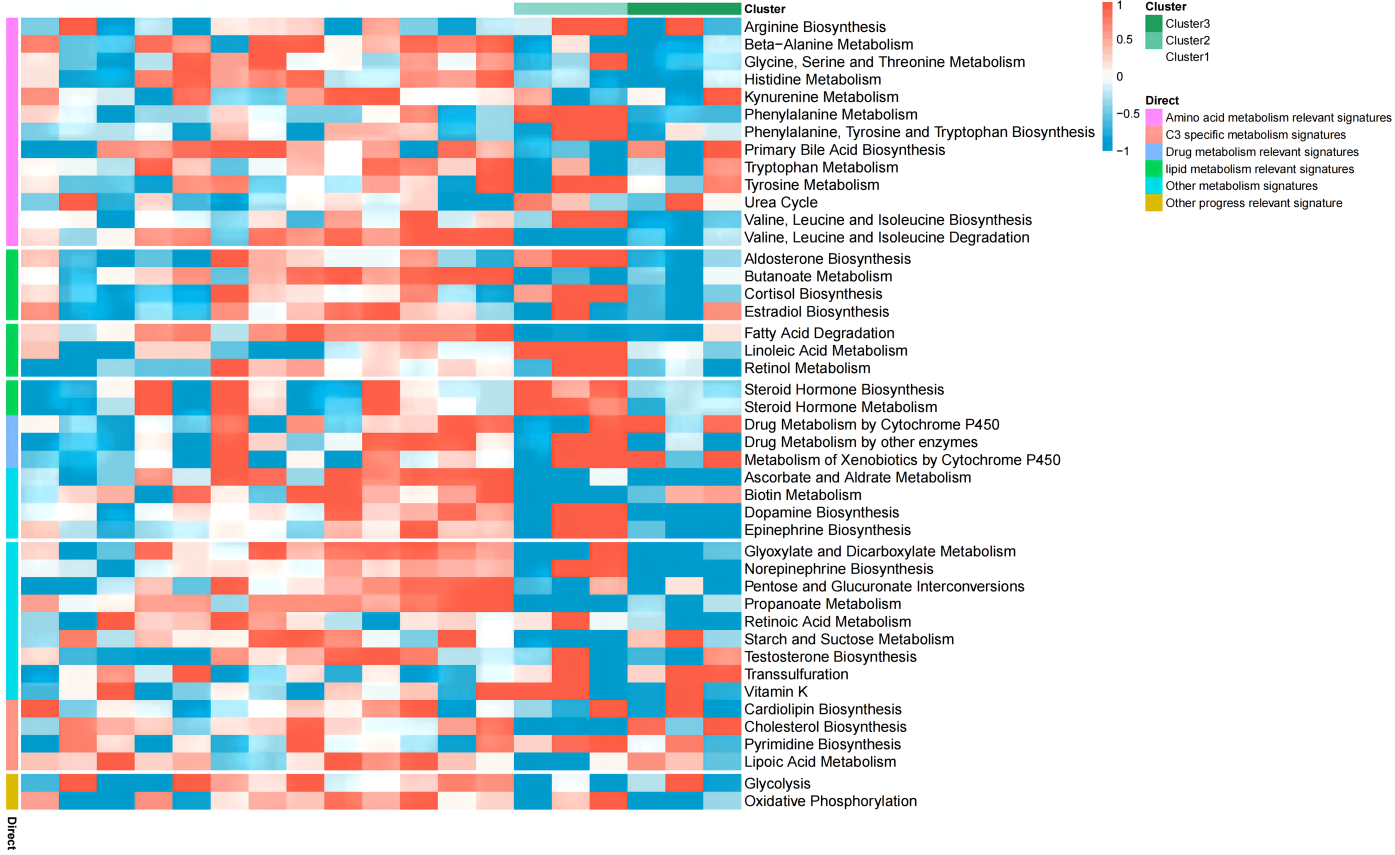
Enrichment of metabolic pathways associated with the three subtypes. Clusters 1 and 2 both show relatively high expression scores with respect to amino acid metabolism, lipid metabolism, drug metabolism, and C3 specific metabolism, and the two clusters principally differed in the latter two. Cluster 3 showed high expression of C3 specific metabolism and amino acid metabolism-related genes, but little or no expression of lipid metabolism-related genes.

We next performed consistent clustering and molecular typing of GSE26887 on the basis of the expression of the identified cuproptosis-related genes (**Figures 3A–C**), which showed that the boundaries of the three subtypes of samples were most clearly defined when K=3, thereby dividing patients with diabetic heart failure into three groups. In addition, we characterized the expression of cuproptosis-related genes in the various subtypes, and found that the vast majority of cuproptosis-related genes were significantly differently expressed among the three subtypes (**Figure 3D**) (**Supplementary Table 4**). The between-group differences in *LOXL2* expression were the largest of the four differentially expressed genes (*GCSH*, *TYR*, *LOXL2*, and *VEGFA*). In addition, we evaluated the relationships of the various subtypes with metabolic pathways (**Figure 4**) (**Supplementary Table 5**). Clusters 1 and 2 had high expression scores with respect to amino acid metabolism, lipid metabolism, drug metabolism, and C3-specific metabolism; with the two differing principally in the latter two. In contrast, cluster 3 showed high expression of C3-specific metabolism and amino acid metabolism-related genes, but little or no expression of lipid metabolism-related genes.

### The differences between the subtypes may be reflected in differences in T-cell infiltration and chemokine family production

**Figure 5:**
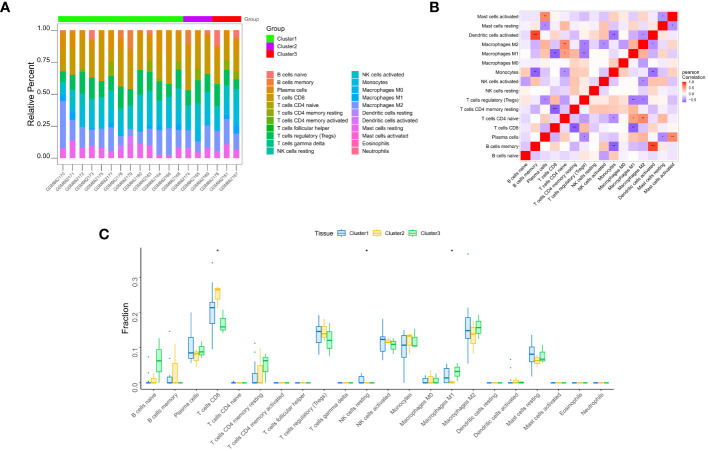
Immune infiltration analysis. **(A)** Differences in the apparent infiltration of immune cells among the three subtypes. **(B)** Heat maps for correlations with the abundances of immune cells for the three subtypes. **(C)** Differences in the immune cell compositions of cardiac samples from patients with the three subtypes. Significant differences in the contents of CD8^+^ T cells, resting NK cells, and M1 macrophages were identified. *, p<0.05; **, p<0.01; ***, p<0.001.

**Figure 6:**
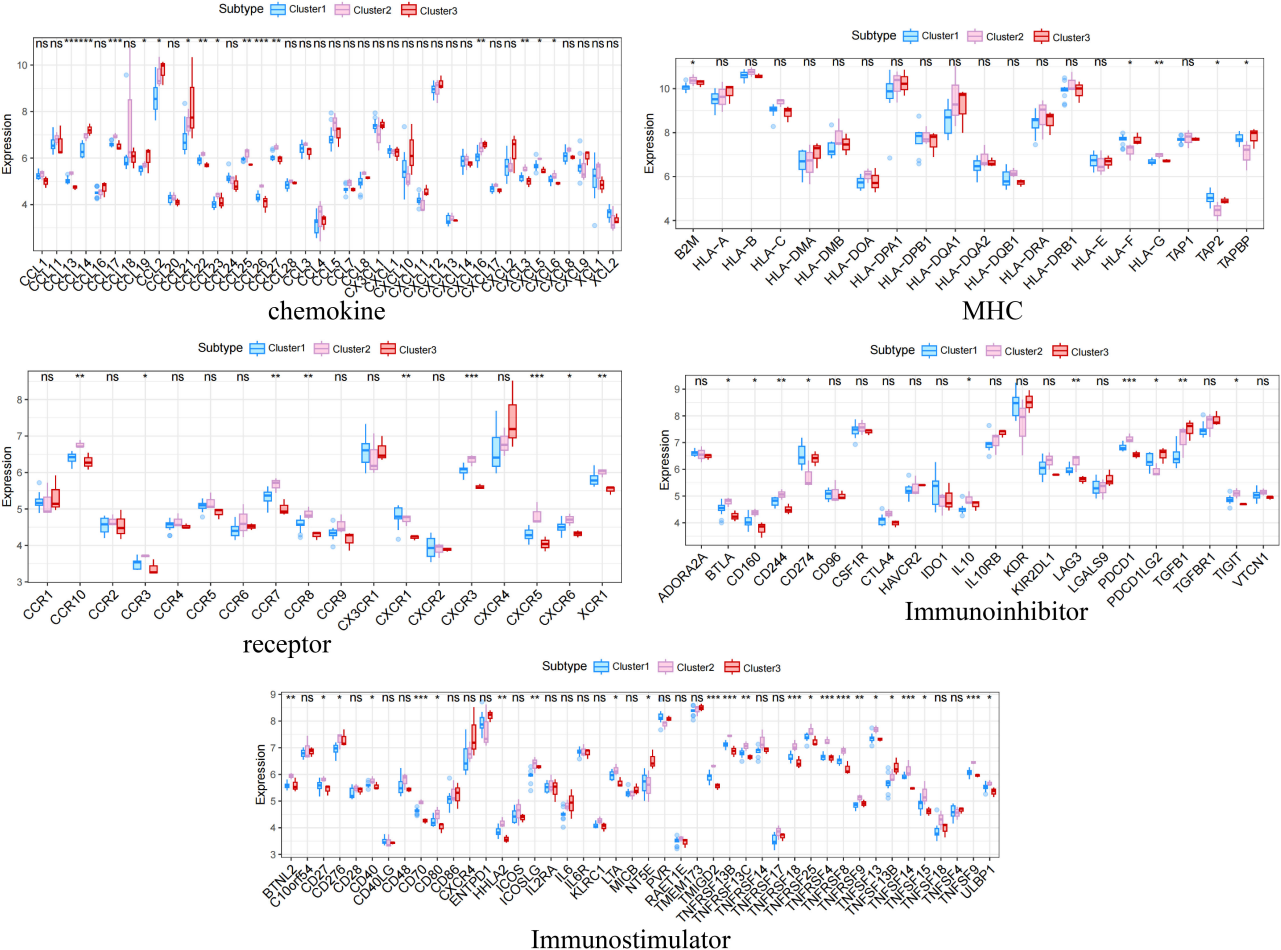
Differences in the expression of immune-related molecules among the three subtypes. Significant differences were found for the receptors XCR1, CXCR6, CXCR5, CXCR4, CCR8, and CCR10; the MHC molecules TAPBP, TAP2, and HLA-DQA1; the chemokines CXCL10, CCL21, CCL2, and CCL18; the immune system promoters NT5E, ENTPD1, and CXCR4; and the immunosuppressive molecules TGF-β1, KDR, and CD274. *, p<0.05; **, p<0.01; ***, p<0.001; ns, no statistically.

The distribution of the levels of immune infiltration and the heatmaps for correlations with immune cell abundances for the various subtypes are shown in **Figures 5A, B**. The immune cell contents of the samples varied substantially. There were several significant relationships among pairs of types of immune cell, including a significant positive correlation between activated dendritic cells and memory B cells, and a significant negative correlation between CD4^+^ memory resting T cells and CD8^+^ T cells. The differences in the immune cell contents of the subtypes are shown in **Figure 5C**; **Supplementary Table 6**. These show significant differences in CD8^+^ T cells, resting NK cell, and M1 macrophages. In addition, the expression of immune-related factors differed among the three subtypes (**Figure 6**) (**Supplementary Tables 7**-**11**): there were significant differences in the expression of the receptors XCR1, CXCR6, CXCR5, CXCR4, CCR8, and CCR10; in the MHC-related molecules TAPBP, TAP2, and HLA-DQA1; in the chemokines CXCL10, CCL21, CCL2, and CCL18; in the immune system promoters NT5E, ENTPD1, and CXCR4; and in the immunosuppressive molecules TGF-β1, KDR, and CD274.

### Results of the screening for key differentially expressed genes between the three subtypes of diabetic heart failure

**Figure 7:**
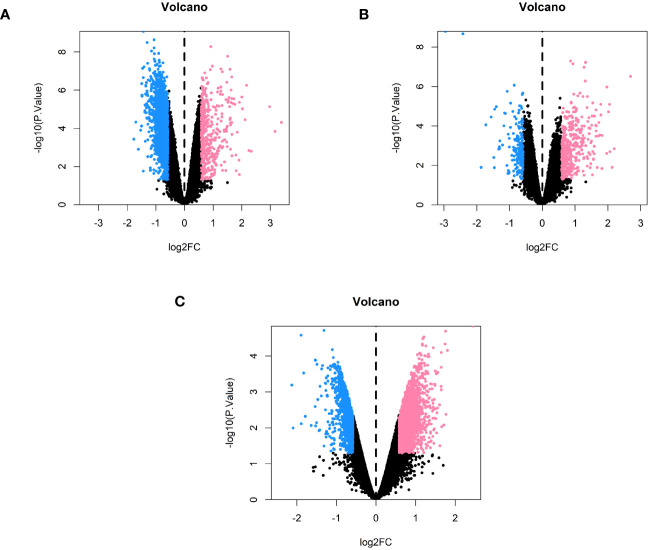
Volcano plots comparing each of the subtypes in pairs. **(A)** Volcano plot of genes that were differentially expressed between subtypes 1 and 2. **(B)** Volcano plot of the genes that were differentially expressed between subtypes 1 and 3. **(C)** Volcano plot of the genes that were differentially expressed between subtypes 2 and 3.

We used the limma package to identify genes that were differentially expressed between the three subtypes, using the conditions |logFC| > 0.585 and *P* < 0.05. A total of 2,313 differentially expressed genes were identified between subtypes 1 and 2, of which 495 were upregulated and 1,818 were downregulated (**Figure 7A**) (**Supplementary Tables 12**-**14**). A total of 573 differentially expressed genes were also identified between subtypes 1 and 3, of which 403 were upregulated and 170 were downregulated (**Figure 7B**) (**Supplementary Tables 15**-**17**). Finally, a total of 4,428 differentially expressed genes were identified between subtypes 2 and 3, of which 2,389 were upregulated and 2,039 were downregulated (**Figure 7C**) (**Supplementary Tables 18**-**20**).

**Figure 8:**
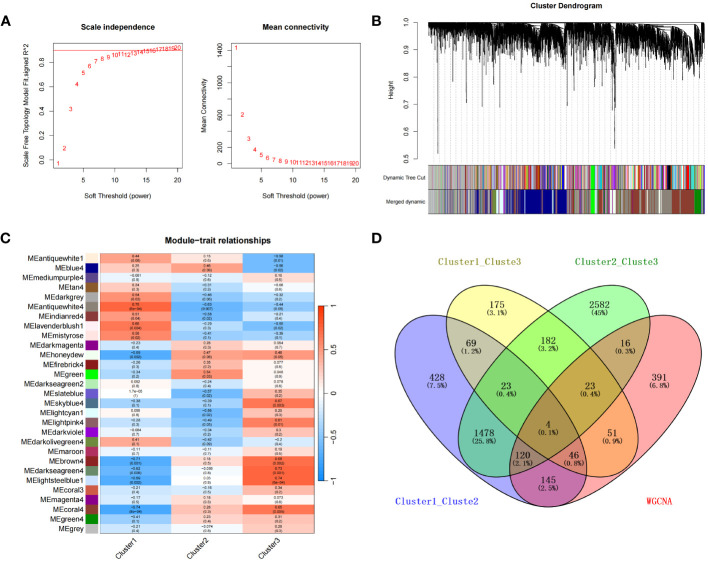
**(A)** Selection of the optimal soft threshold (power). **(B)** Recognition of gene modules. **(C)** Heat map of the relationships between gene modules and clinical traits for the three subtypes. **(D)** Four overlapping were genes identified for the three subtypes using a Venn diagram.

To characterize the co-expression network for genes characterizing the diabetic heart failure samples, we constructed a WGCNA network using a soft threshold β of 17 (**Figure 8A**) and then identified gene modules using the TOM matrix. A total of 29 gene modules were identified using this analysis (**Figure 8B, C**), which were: antiquewhite1 (14), antiquewhite4 (796), blue4 (1,100), brown4 (32), coral3 (21), coral4 (942), darkgrey (41), darkmagenta (36), darkolivegreen4 (118), darkseagreen2 (14), darkseagreen4 (28), darkviolet (24), firebrick4 (24), green (85), green4 (213), grey (690), honeydew (222), indianred4 (25), lavenderblush1 (15), lightcyan1 (216), lightpink4 (143), lightsteelblue1 (34), magenta4 (53), maroon (29), mediumpurple4 (21), mistyrose (14), skyblue4 (21), slateblue (13), and tan4 (16); with the closest correlation being for the MEantiquewhite4 module (cor=0.75, *p*=6e−04). Subsequently, we interrogated the intersection of the differentially expressed genes among the various subtypes with the genes in the MEantiquewhite4 module using Venn plots, which showed the expression of four genes (*BCO2*, *HSDL2*, *CORIN*, and *SNORA80E*) (**Figure 8D**) (**Supplementary Table 21**). These were regarded as the key genes in our subsequent analyses.

### Correlations between the expression of the key genes and that of immune-related genes

**Figure 9:**
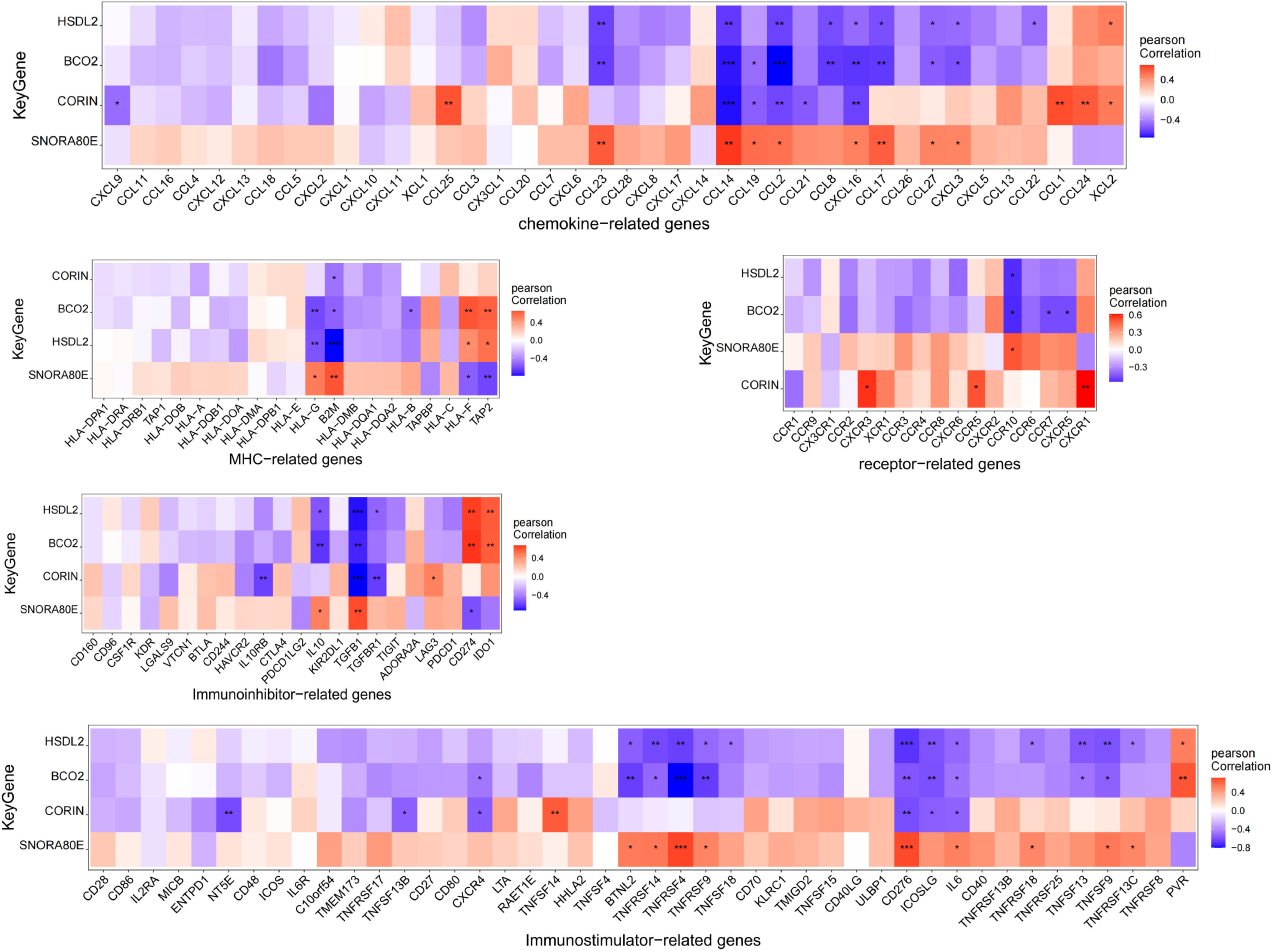
Correlations between the expression of the four key genes and immune-related genes. The expression of *HSDL2*, *BCO2*, and *CORIN* negatively correlated with that of multiple immune-related genes, whereas the expression of *SNORA80E* showed positively correlations. *, p<0.05; **, p<0.01; ***, p<0.001.

We next analyzed the relationships of the expression of *BCO2*, *HSDL2*, *CORIN*, and *SNORA80E* with that of various immune-related genes, including those encoding immunosuppressive factors, immunostimulatory factors, chemokines, and receptors. The correlations obtained suggested that these key genes are closely associated with immune-related factors and play important roles in the immune microenvironment. Specifically, the expression of *HSDL2*, *BCO2*, *CORIN* negatively correlated with that of multiple immune-related genes; whereas the expression of *SNORA80E* positively correlated with that of a variety of immune-related genes (**Figure 9**) (**Supplementary Tables 22**-**26**).

### lncRNA-miRNA-mRNA networks involving the identified key genes

**Figure 10:**
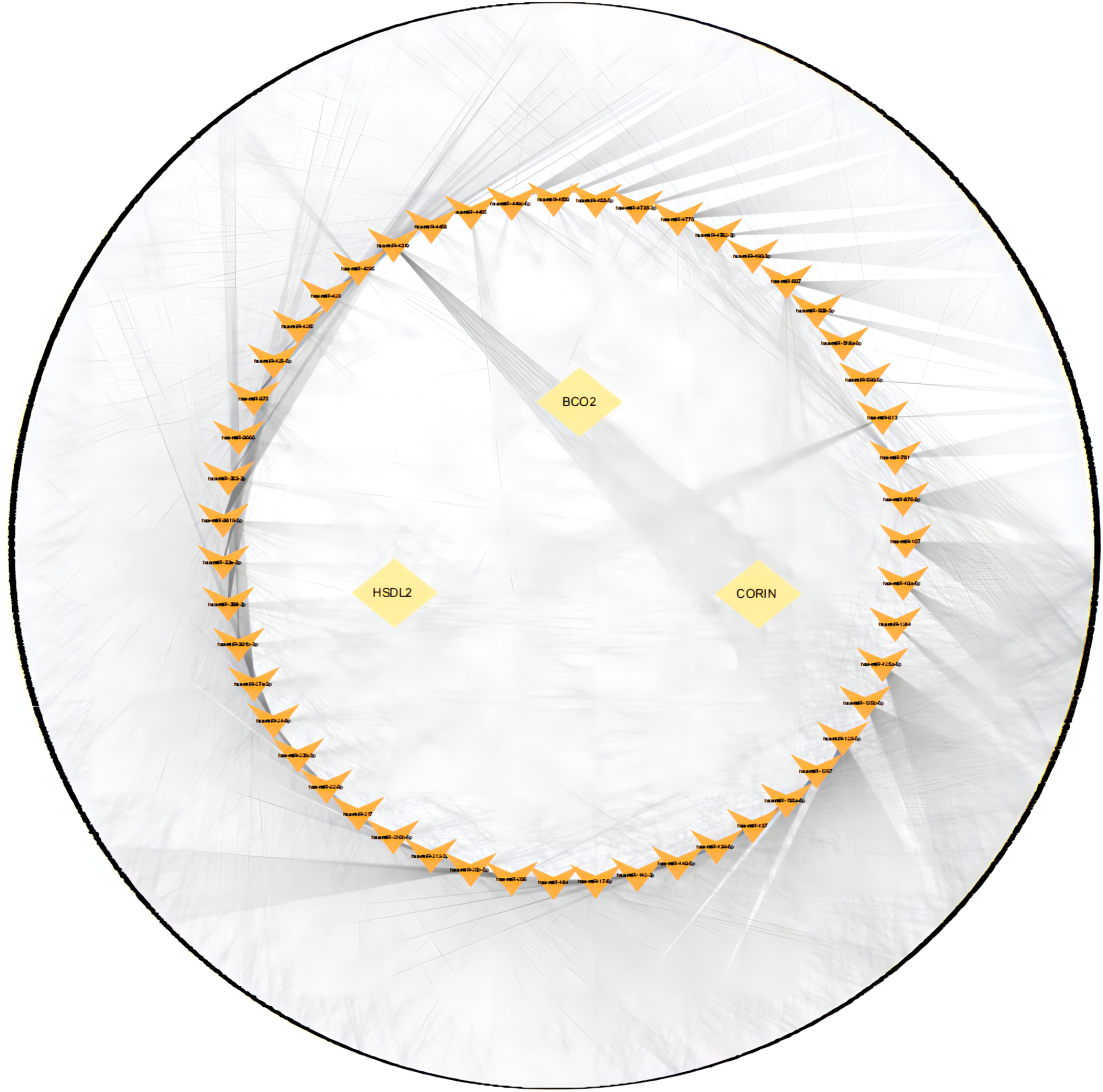
Competitive endogenous RNA network for the identified key genes. The inner ring represents the key genes, the middle ring represents microRNAs, and the outer ring represents long non-coding RNAs.

To construct an lncRNA-miRNA-mRNA network for the identified genes, we performed miRNA target prediction for the four key genes using the mircode database, which identified 51 key gene-related target miRNAs. Subsequently, we performed lncRNA target prediction using 21 of these miRNAs using the mircode database, which predicted a total of 3,849 lncRNAs and 7,642 lncRNA-miRNA-mRNAs relationships. We used these to construct a predicted lncRNA-miRNA-mRNA network, which is visualized using cytoscape in **Figure 10**; **Supplementary Table 27**.

### Results of the GSEA pathway enrichment analysis for the four identified key genes

**Figure 11:**
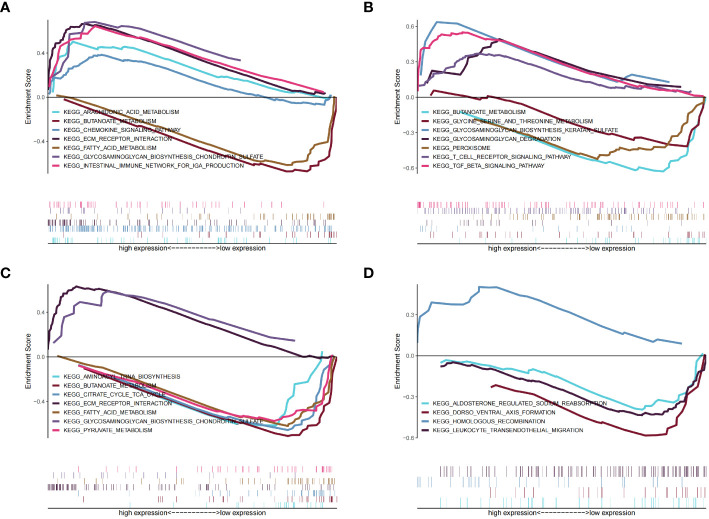
Results of the gene set enrichment analysis (GSEA) for the four identified key genes. **(A)** *BCO2*. **(B)** *CORIN*, **(C)** *HSDL2*. **(D)** *SNORA80E*.

We next sought to identify the specific signaling pathways associated with the four key genes to explore the potential means whereby the key genes are involved in cardiomyocyte pathology in patients with diabetes, and consequently in the development of heart failure. GSEA showed that *BCO2* was associated with arachidonic acid metabolism, butanoate metabolism, and other pathways (**Figure 11A**) (**Supplementary Table 28**); *CORIN* was associated with pathways such as peroxisomes and TGF-β signaling (**Figure 11B**) (**Supplementary Table 29**); *HSDL2* was associated with pathways such as fatty acid metabolism and pyruvate metabolism (**Figure 11C**) (**Supplementary Table 30**); and *SNORA80E* was associated with dorsoventral axis formation and homologous recombination (**Figure 11D**) (**Supplementary Table 31**). These findings suggest that the identified genes may influence disease progression through these pathways.

### The transcription factor MECOM may be an important regulator of the identified key genes, and thereby affect cardiomyocytes in diabetic heart failure

**Figure 12:**
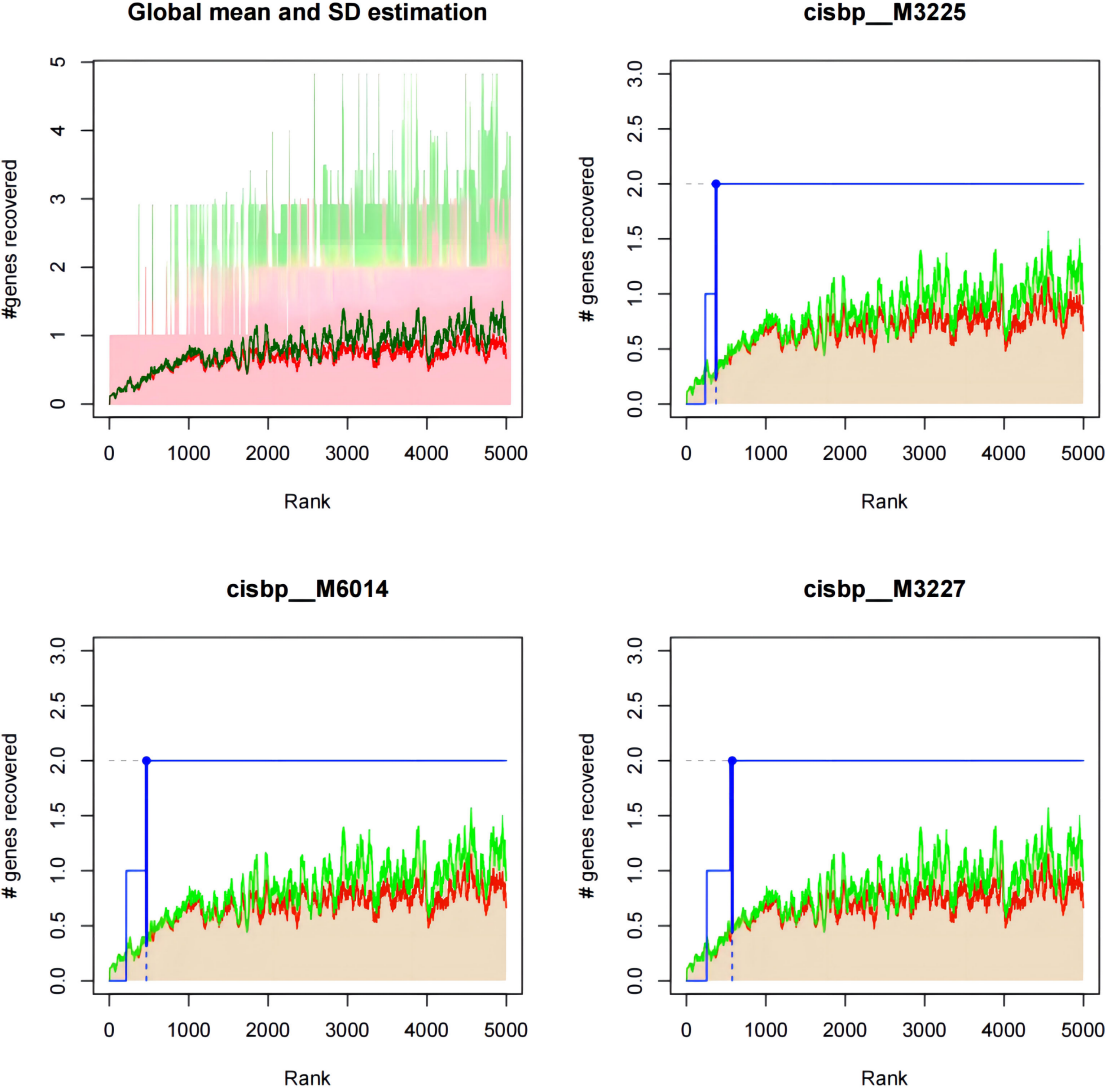
Motif-enrichment associated with the identified key genes. Cisbp:M3225, cisbp:M6014, and cisbp:M3227 were the three motifs with the highest enrichment scores.

We found that the expression of the four key genes was regulated by common mechanisms, including by multiple transcription factors. Therefore, we performed enrichment analysis for these transcription factors using cumulative recovery curves. Motif-TF annotation and selection analysis of the genes showed that the motif with the highest normalized enrichment score (NES: 8.96) was cisbp:M3225, and the main transcription factor associated with this motif was MECOM. All the enriched motifs for the key genes and the corresponding transcription factors are shown in **Figure 12**; **Supplementary Table 32**.

### Correlations of the expression of the identified key genes with that of other diabetic heart failure-associated regulatory genes

**Figure 13:**
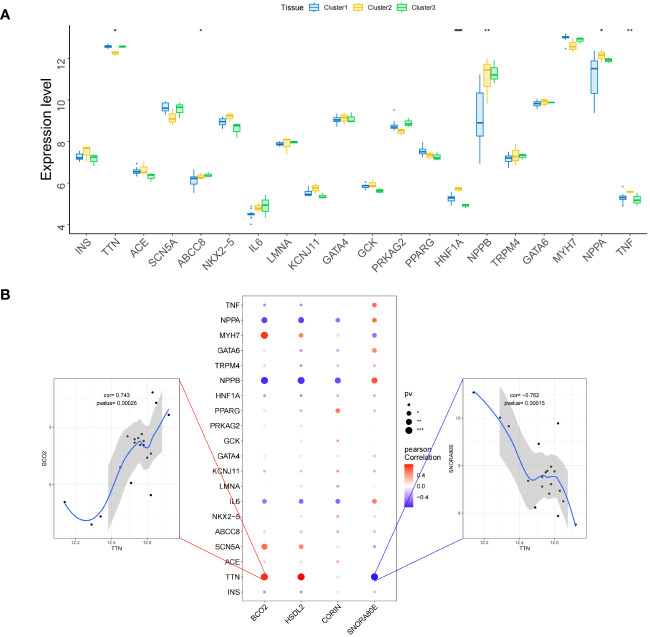
Relationships between the identified key genes and diabetic heart failure-related genes in the validation set. **(A)** Inter-group differences in the expression of genes associated with diabetic heart failure. **(B)** Correlations of the expression of the key genes with that of genes associated with diabetic heart failure. Red represents a positive correlation and blue represents a negative correlation. *, p<0.05; **, p<0.01; ***, p<0.001.

We obtained a list of diabetic heart failure-related genes from the GeneCards database (https://www.genecards.org/). We then listed the expression levels of the top 20 genes with respect to the Relevance score and compared the expression of the disease-related genes between the groups. We found that the expression of *TTN*, *ABCC8*, *HNF1A*, *NPPB*, *NPPA*, and *TNF* differed between the two groups of patients (**Figure 13A**) (**Supplementary Table 33**). In addition, the expression of the four key genes significantly correlated with that of several disease-related genes (**Figure 13B**) (**Supplementary Table 34**). For example, *BCO2* and *TTN* expression significantly positively correlated (r = 0.743), and *SNORA80E* and *TTN* expression significantly negatively correlated (r = −0.762).

## Discussion

Cuproptosis has recently been identified as a mode of cell death, and it has been demonstrated that a decrease in the plasma concentration of copper ions promotes oxidative stress, which in turn induces type 2 diabetes mellitus (22). Given that there are also high copper ion concentrations in the myocardial tissue of patients with heart failure, it may be that intracellular copper ion accumulation occurs in the cardiomyocytes of patients with diabetes mellitus-associated heart failure. In the present study, we have used a bioinformatics approach to identify biomarkers of cuproptosis in diabetic heart failure: GCSH, TYR, LOXL2, and VEGFA. Furthermore, by analysis of expression data relating to subtypes of cuproptosis in diabetic heart failure, we have identified four key genes: *HSDL2*, *BCO2*, *CORIN*, and *SNORA80E*. *GCSH* and *TYR* expression was low in disease samples, whereas that of *LOXL2*, and *VEGFA* was high. In addition, the expression of the four key genes significantly correlated with that of immune-related genes, and all four were found to be associated with multiple signaling pathways, and they all appeared to regulated by the transcription factor MECOM.

The most notable of the key genes is *LOXL2*, which encodes lysyl oxidase-like 2, an extracellular copper-dependent enzyme that plays a central role in fibrosis by catalyzing the cross-linking and deposition of collagen. The overexpression of this gene leads to the excessive deposition of collagen and other components of the extracellular matrix (ECM), thereby causing fibrosis in the heart, blood vessels, and other organs, which ultimately leads to the development of a variety of cardiovascular diseases. Copper ions are required for LOXL2 enzyme activity, and the protein also contains a lysine tyrosine quinone region (LTQ) that binds cofactors, providing a theoretical basis for the LOXL2 protein to be a target for cuproptosis-related diseases. Dongiovanni et al. found that insulin resistance is positively associated with high LOXL2 expression and the development of fibrosis in patients with T2DM; and consistent with this, the silencing of FoxO1 normalizes LOXL2 expression, thereby ameliorating the fibrosis of InsR+/−(insulin receptor (InsR) haploinsufficiency) MCD(methionine-choline deficient)-fed mice (23). Johnson et al. also showed that serum LOXL2 concentration and myocardial *LOXL2* mRNA expression of mice with diabetic heart failure are high, whereas the expression of the profibrotic protein COL1A in LOXL2-deficient H9C2 cardiomyocytes exposed to a high-glucose environment is low (24). Taken together, these results suggest that LOXL2 plays an important role in myocardial fibrosis in diabetes, which occurs secondary to the high-glucose environment and is mediated through FoxO1.

Another study also showed that hyperactivation of the PI3K/Akt/FoxO1/LOXL2 pathway promotes the development of myocardial fibrosis in the cardiomyocytes of patients with diabetes (25). Furthermore, it is noteworthy that miR-27b-3p inhibitors have been shown to impair the anti-LOXL2 activity of human umbilical cord mesenchymal stem cell exosomes (MSC-ex), thereby reducing their antifibrotic effects, whereas miR-27b-3p overexpression promotes MSC-ex-mediated YAP/LOXL2 inhibition (26). This suggests that MSC-ex may reduce LOXL2 expression by downregulating YAP expression via miR-27b-3p. In addition, it has been shown that MSC-ex promotes pancreatic β-cell proliferation, ameliorates insulin resistance, and reduces oxidative stress-induced damage (27). Therefore, taking all these findings together, it is reasonable to hypothesize that in the cardiomyocytes of patients with diabetes, the high-glucose environment stimulates the hyperactivation of the PI3K/Akt/FoxO1/LOXL2 pathway and the development of myocardial fibrosis, and consequently induces heart failure. During this process, copper ions concentrations increase in cardiomyocytes to maintain LOXL2 enzyme activity, and cardiomyocytes release exosomes containing miR-27b-3p, which reduce the activation of the YAP/LOXL2 pathway, having a compensatory anti-fibrotic effect.

It has previously been shown that in mouse macrophages, ERK1/2 upregulated the expression of GCSH, thereby promoting the production of AGEs (28), which are involved in the pathogenesis of diabetes-related atherosclerosis. TYR-encoded enzymes require copper ions for their dual catalytic activity of tyrosine hydroxylase and dopa oxidase (29). TYR expression is positively associated with the incidence of cardiovascular events in patients with diabetes (30), and these enzymes activate the IL-6/STAT3 pathway, thereby inhibiting mitochondrial function (31), as well as regulating the nitration of caveolin-3, which causes insulin resistance in diabetic mice (32).

The cardiac expression of *VEGFA* was found to significantly positively correlate with that of *LOXL2* in the present study, which may be associated with the stimulation of microangiogenesis in response to LOXL2-induced myocardial fibrosis. In the presence of hyperglycemia, the oxygen-carrying capacity of erythrocytes is lower than normal, and the resulting hypoxia activates HIF-lα, which causes an increase in VEGFA production (33). VEGFA participates in the SIRTI/FOXO3a/MnSOD pathway and inhibits mitochondrial oxidative stress, ameliorating cardiac dysfunction in rats with heart failure (34). Notably, cysteine oxidation of the copper transporter CTR1 drives VEGFR2 signaling and angiogenesis (35). This suggests that the copper transporter CTR1 may indirectly stimulate the upregulation of VEGFA in patients with diabetes mellitus-associated heart failure, which is cardioprotective.

*HSDL2* was found to be associated with fatty acid metabolism in the present study, and indeed the silencing of HSDL2 results in the inhibition of lipid metabolism, and therefore lower availability of the principal source of energy for cardiomyocytes, the oxidation of long-chain fatty acids (36), which causes heart failure. Low *BCO2* expression causes cardiac retinoic acid deficiency and impaired metabolic flexibility in mice, and this is associated with impaired activation of the PDK4/PDH pathway (37). In addition, *in vitro* studies have shown that BCO2 activity is 7% higher in medium containing 5 mM Cu^2+^ (38), suggesting that copper ions may be involved in various pathophysiological processes in cardiomyocytes through an effect on BCO2 activity, and thereby the PDK4/PDH pathway, which affects myocardial energy metabolism.

CORIN is a type II transmembrane serine protease that is expressed on cardiomyocyte membranes (39). Its defective expression in mice results in hypertension and cardiac hypertrophy, which leads to impaired cardiac function through abnormal activation of the TGF-β signaling pathway (40) and pro-ANP (41). The homologous recombination associated with the high SNORA80E expression identified in the present study may involve ShcA, encoding Src homologous-collagen homolog, and the epigenetically sustained expression of ShcA in diabetes-associated atherosclerosis is associated with macrophage instability and the regression of atherosclerosis (42).

In the present analysis of the relationships between the expression of the identified key genes and that of immune-related genes, we found that *HSDL2*, *BCO2*, and *CORIN* expression significantly negatively correlated with the expression of the chemokine genes *CCL14*, *CCL19*, *CCL2*, *CCL21*, *CCL8*, and *CXCL16*; whereas the opposite relationships were identified for *SNORA80E*. The CCL family is directly involved in oxidative stress injury to cardiomyocytes and contributes to cardiomyocyte fibrosis by upregulating the Raf kinase inhibitor protein (RKIP), which promotes fibrosis in cardiomyocytes (43). In a further analysis of the relationships of the expression of key genes with that of immunomodulatory factors and receptors, we found that *HSDL2*, *BCO2*, and *CORIN* expression negatively correlated with the expression of TGF-β family genes, tumor necrosis factor superfamily (TNFSF) genes, *HLA*-G, *B2M*, and *CCR10*, whereas the opposite relationships were identified for *SNORA80E*. These findings suggest that the role of the key genes in the cellular microenvironment might be associated with CD8^+^ T cells, resting NK cells, and M1 macrophages. In a previous study, snoRNAs were shown to remodel the cellular microenvironment by modulating the GAB2/AKT/mTOR signaling pathway, blocking immune checkpoints, and reducing cardiac infiltration with CD8^+^ T cells (44); and this finding is consistent with the low expression of *SNORA80E* and the infiltration with CD8^+^ T cells identified in the present study. Taking these results together, we can speculate that the product of the cuproptosis-related gene *SNORA80E* alters the immune microenvironment of cardiomyocytes via the mTOR-related signaling pathway and that CD8^+^ T cells are the predominant infiltrating cell type, contributing to the onset and progression of diabetic heart failure.

We also identified the miRNAs that regulate HSDL2, BCO2, and CORIN expression, as well as the lncRNAs regulated by these miRNAs, and constructed a ceRNA regulatory network. Of the identified mRNAs, miR-375 directly targets PI3K molecules and increases their expression (45). As described above, PI3K is an upstream regulator of LOXL2, suggesting that this miR-375 may be involved in LOXL2-induced myocardial fibrosis. However, it has previously been shown that the activation of the miR-375/FOXF1 axis in exosomes inhibits fibrosis (46). Thus, a number of miRNAs are associated with diabetic heart failure and cuproptosis, and miR-375 likely interacts with LOXL2 and participates in the development of myocardial fibrosis, while also having an anti-fibrotic effect in combination with miR-27b-3p in exosomes.

Finally, we analyzed the relationships of the expression of the key genes (*HSDL2*, *BCO2*, *CORIN*, and *SNORA80E*) with that of transcription factors and regulatory genes in samples from patients with diabetes-associated heart failure. The transcription factor MECOM was found to be closely associated with the key genes, according to motif enrichment analysis. MECOM is a transcription factor that is closely associated with the zinc finger structure of proteins (47). Recent single-cell RNA sequencing studies have shown that MECOM is a regulator of the human cardiac endothelium (48) and is involved in epigenetic regulation (49). This suggests that cuproptosis-related genes may influence the development of diabetes-associated heart failure by altering the structure of functional proteins in cardiomyocytes via MECOM, and these effects may be transmitted epigenetically. We selected the top 20 genes according to the correlation index and analyzed their expression, finding a significant positive correlation between the expression of *BCO2* and *TTN* and a significant negative correlation between the expression of *SNORA80E* and *TTN*. A previous *in vitro* study showed that mutations or aberrant expression of *TTN* can cause diabetic cardiomyopathy by truncating the titin protein and causing haploinsufficiency (50). Thus, in patients with diabetes mellitus in combination with heart failure, BCO2 and SNORA80E may have direct or indirect effects on TTN, and each gene product is likely to regulate the expression of the other as part of the mechanism of cuproptosis.

In summary, in the present study, we have shown that the cuproptosis gene *LOXL2* is likely to be involved in the development of heart failure in patients with diabetes, with a putative mechanism as follows. First, the high-glucose environment upregulates the PI3K/Akt/FoxO1/LOXL2 pathway, leading to the development of myocardial fibrosis and then heart failure. During this process, copper ions accumulate in cardiomyocytes and maintain LOXL2 enzyme activity. At this time, cardiomyocytes release exosomes, and miR-27b-3p in these exosomes reduce the activation of the YAP/LOXL2 pathway, which has a compensatory anti-fibrotic role. Second, the pathogenesis involves T-cell infiltration, exosomes, and the participation of miRNAs, of which miR-375 may be upregulated in the exosomes and act as a trans-regulator of LOXL2. Third, the transcription factor MECOM is involved in altering the structure of proteins, and thereby their cellular function. Finally, the cuproptosis-related genes *BCO2* and *SNORA80E* may jointly regulate the expression of TTN, and thereby the cuproptosis of cells.

In the present study, we have predicted the relationships of DNA, RNA, immune cells, and immunological molecules with diabetes mellitus-associated heart failure using a bioinformatic approach. This has enabled us to quickly identify potentially relevant pathogenic factors. However, there are still some limitations in this study. First, given the small amount of data used, it was not possible to perform further phenotypic screening with respect to the type of diabetes or heart failure, or to explore the causal relationship between cuproptosis-related genes and heart failure secondary to diabetes. Second, bioinformatic analysis methods cannot determine whether the two factors involved are directly or indirectly related, which requires further cell or animal experiments to verify.

## Data availability statement

The dataset (GSE26887) analyzed in this study were downloaded from the Gene Expression Omnibus (GEO) database (https://www.ncbi.nlm.nih.gov/geo/). The list of diabetic heart failure-related genes which was selected as the validation set was downloaded from the GeneCards database (https://www.genecards.org/). Cuproptosis-associated genes were identified from the previous publication “Identification of cuproptosis-related subtypes and development of a prognostic signature in colorectal cancer.” (DOI: 10.1038/s41598-022-22300-2).

## Author contributions

JC: Conceptualization, Data curation, Formal analysis, Methodology, Project administration, Resources, Software, Supervision, Validation, Visualization, Writing – original draft, Writing – review & editing. XY: Data curation, Formal analysis, Funding acquisition, Supervision, Writing – review & editing. WL: Conceptualization, Investigation, Writing – review & editing. YL: Conceptualization, Investigation, Writing – review & editing. RL: Conceptualization, Investigation, Writing – review & editing. XC: Conceptualization, Investigation, Writing – review & editing. BY: Conceptualization, Investigation, Writing – review & editing. BX: Conceptualization, Investigation, Writing – review & editing. JL: Funding acquisition, Supervision, Writing – review & editing.

## Supplementary material

Supplementary Table 1 The list of diabetic heart failure-related genes obtained from the Gene Expression Omnibus (GEO) database.

Supplementary Table 2 Heat map of the differentially expressed genes associated with cuproptosis in samples from healthy controls.

Supplementary Table 3 Heat map of the differentially expressed genes associated with cuproptosis in samples from patients with diabetic heat failure.

Supplementary Table 4 The list of GEO Samples (GSM) of each cluster.

Supplementary Table 5 The correlationship with metabolic pathways of each cluster.

Supplementary Table 6 The differences in the immune cell contents of the subtypes.

Supplementary Table 7 The differences in chemokine among subtypes.

Supplementary Table 8 The differences in immunoinhibitor among subtypes.

Supplementary Table 9 The differences in immunostimulator among subtypes.

Supplementary Table 10 The differences in MHC among subtypes.

Supplementary Table 11 The differences in immune receptor among subtypes.

Supplementary Table 12 The list of 2,313 differentially expressed genes identified between subtypes 1 and 2.

Supplementary Table 13 The list of 495 upregulated differential genes identified between subtypes 1 and 2.

Supplementary Table 14 The list of 1818 downregulated differential genes identified between subtypes 1 and 2.

Supplementary Table 15 The list of 573 differentially expressed genes identified between subtypes 1 and 3.

Supplementary Table 16 The list of 403 upregulated differential genes identified between subtypes 1 and 3.

Supplementary Table 17 The list of 170 downregulated differential genes identified between subtypes 1 and 3.

Supplementary Table 18 The list of 4428 differentially expressed genes identified between subtypes 2 and 3.

Supplementary Table 19 The list of 2389 upregulated differential genes identified between subtypes 2 and 3.

Supplementary Table 20 The list of 2039 downregulated differential genes identified between subtypes 2 and 3.

Supplementary Table 21 The intersection of the differentially expressed genes among the various subtypes with the genes in the MEantiquewhite4 module.

Supplementary Table 22 The correlations between the expression of the key genes and that of chemokine-related genes.

Supplementary Table 23 The correlations between the expression of the key genes and that of immunoinhibitor-related genes.

Supplementary Table 24 The correlations between the expression of the key genes and that of immunostimulator-related genes.

Supplementary Table 25 The correlations between the expression of the key genes and that of MHC-related genes.

Supplementary Table 26 The correlations between the expression of the key genes and that of immune receptor-related genes.

Supplementary Table 27 The lncRNA-miRNA-mRNA network.

Supplementary Table 28 The GSEA enrichment analysis of BCO2.

Supplementary Table 29 The GSEA enrichment analysis of CORIN.

Supplementary Table 30 The GSEA enrichment analysis of HSDL2.

Supplementary Table 31 The GSEA enrichment analysis of SNORA80E.

Supplementary Table 32 The enriched motifs for the key genes and the corresponding transcription factors.

Supplementary Table 33 The correlations of the expression of the identified key genes with that of other diabetic heart failure-associated regulatory genes.

Supplementary Table 34 The correlationship between the expression of the four key genes and that of several disease-related genes.
