# Supplementary figures and images for: Potential molecular and cellular mechanisms of the effects of cuproptosis-related genes in the cardiomyocytes of patients with diabetic heart failure: a bioinformatics analysis
Source: Front Endocrinol (Lausanne). 2024 May 31;15:1370387. doi: 10.3389/fendo.2024.1370387 (PMC11176466; doi:10.3389/fendo.2024.1370387)

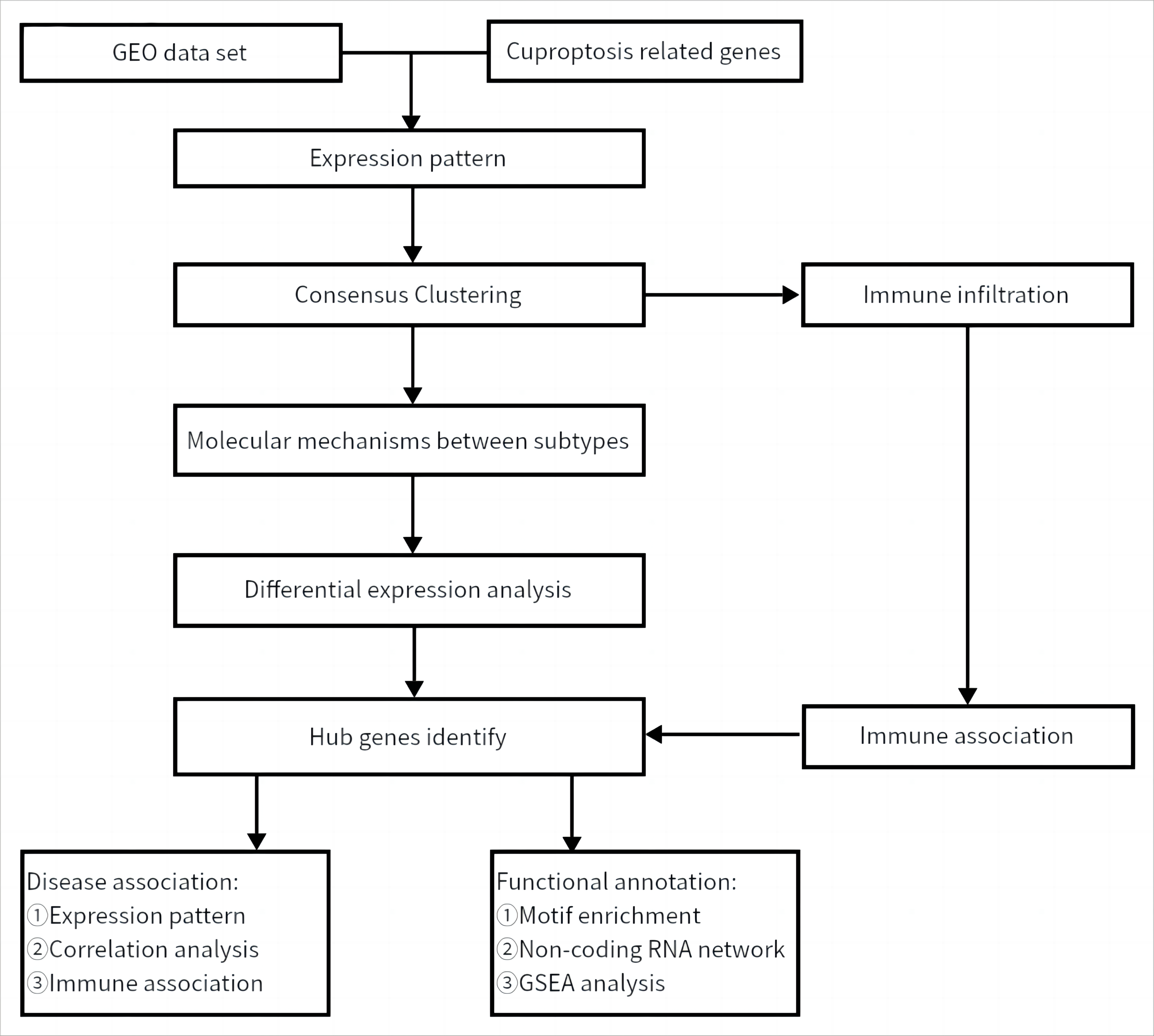

Supplement: Supplementary file 1 [file Image_1.png]
